# Observation of an E2 (Ubc9)-homodimer by crystallography

**DOI:** 10.1016/j.dib.2016.02.015

**Published:** 2016-02-12

**Authors:** Aileen Y. Alontaga, Nigus D. Ambaye, Yi-Jia Li, Ramir Vega, Chih-Hong Chen, Krzysztof P. Bzymek, John C. Williams, Weidong Hu, Yuan Chen

**Affiliations:** Department of Molecular Medicine, Beckman Research Institute of the City of Hope, 1450 East Duarte Road, Duarte, CA 91010, United States

**Keywords:** SUMO, Poly-SUMO chain, RWD, Ubiquitin, Ubc9, E1, Ubiquitin-like, Modifications

## Abstract

Post-translational modifications by the small ubiquitin-like modifiers (SUMO), in particular the formation of poly-SUMO-2 and -3 chains, regulates essential cellular functions and its aberration leads to life-threatening diseases (Geoffroy and Hay, 2009) [1]. It was shown previously that the non-covalent interaction between SUMO and the conjugating enzyme (E2) for SUMO, known as Ubc9, is required for poly-SUMO-2/3 chain formation (Knipscheer et al., 2007) [2]. However, the structure of SUMO-Ubc9 non-covalent complex, by itself, could not explain how the poly-SUMO-2/3 chain forms and consequently a Ubc9 homodimer, although never been observed, was proposed for poly-SUMO-2/3 chain formation (Knipscheer et al., 2007) [2]. Here, we solved the crystal structure of a heterotrimer containing a homodimer of Ubc9 and the RWD domain from RWDD3. The asymmetric Ubc9 homodimer is mediated by the N-terminal region of one Ubc9 molecule and a surface near the catalytic Cys of the second Ubc9 molecule (Fig. 1A). This N-terminal surface of Ubc9 that is involved in the homodimer formation also interacts with the RWD domain, the ubiquitin-fold domain of the SUMO activating enzyme (E1), SUMO, and the E3 ligase, RanBP2 (Knipscheer et al., 2007; Tong et al.. 1997; Tatham et al., 2005; Reverter and Lima, 2005; Capili and Lima, 2007; Wang et al., 2009, 2010; Wang and Chen, 2010; Alontaga et al., 2015) [2], [3], [4], [5], [6], [7], [8], [9], [10]. The existence of the Ubc9 homodimer in solution is supported by previously published solution NMR studies of rotational correlation time and chemical shift perturbation (Alontaga et al., 2015; Yuan et al., 1999) [10], [11]. Site-directed mutagenesis and biochemical analysis suggests that this dimeric arrangement of Ubc9 is likely important for poly-SUMO chain formation (Fig. 1B and C). The asymmetric Ubc9 homodimer described for the first time in this work could provide the critical missing link in the poly-SUMO chain formation mechanism. The data presented here are related to the research article entitled, “RWD domain as an E2 (Ubc9) interaction module” (Alontaga et al., 2015) [10]. The data of the crystal structure has been deposited to RCSB protein data bank with identifier: 4Y1L.

**Specifications Table**

**Table:** 

Subject area	*Biological Chemistry/Structure Biology*
More specific subject area	*Ubiquitin-like modifications, SUMO, RWD*
Type of data	*Table, figures, structure coordinates, gel images, NMR data*
How data was acquired	*X-Ray diffraction, NMR spectrometer, biochemical assays*
Data format	*Raw and analyzed*
Experimental factors	*None applied*
Experimental features	*Protein expression and purification, Isotope labeling of proteins with*^13^C *and*^15^N*, NMR spectra collection and chemical shift perturbation analysis of protein-protein interactions, identification of crystal growth condition, crystal diffraction, structure determination and refinement, and biochemical assays*
Data source location	*Beckman Research Institute of the City of Hope, Duarte, CA, USA*
Data accessibility	*The X-ray crystal structure data is available at RCSB protein data bank with PDB identifier 4Y1L* (http://www.rcsb.org/pdb/explore/explore.do?structureId=4Y1L)

**Value of the data** 

• Provides the first reported E2 homodimer crystal structure.
• Establishes a role for the evolutionary conserved RWD domain.
• The asymmetric Ubc9 homodimer described in this work could provide the critical missing link in the poly-SUMO chain formation mechanism.

## 1 Data, experimental design, materials and methods

### 1.1 Protein expression and purification

The recombinant proteins contained His_6_-tag and were expressed in *Escherichia coli* and purified by Ni-NTA-column. Protein purity was greater than 90% as estimated by Coomasie-stained SDS gel.

### 1.2 NMR sample preparation and experiments

All NMR spectra were acquired at 25 °C on a Bruker Avance spectrometer equipped with a cryo-probe and operating at a 600-MHz ^1^H frequency.

### 1.3 Biochemical assays

All SUMO conjugation assays were conducted in a mixture that contained 5 mM ATP and assay buffer (20 mM HEPES, pH 7.5, 50 mM NaCl, 5 mM MgCl_2_, and 0.1% TWEEN) unless otherwise stated. Assay reactions were incubated at 37 °C and were stopped by addition of SDS loading buffer. Samples were resolved on 4−12% Bis-Tris NuPAGE SDS-PAGE gels (Invitrogen), and the polypeptide bands were visualized with SimplyBlue SafeStain (Invitrogen). To investigate the effect of Ubc9 WT and mutant on poly-SUMO chain formation, a mixture containing 50 mM Tris pH 7.6, 150 mM NaCl, 5 mM MgCl_2_, 0.05% Triton X, 1 mM DTT, E1 (0.2 µM), SUMO-2/3 (100 µM), 5 mM ATP were incubated with Ubc9 WT or the Y134A (5 and 10 µM) for 4 h before stopping it with reducing SDS loading buffer. Sp100-SUMO conjugation assay contained 0.25 μM E1, 0.25 μM Ubc9, 2 μM of the M-IR2 domain of RanBP2 [4] (referred as RanBP2), 15 μM GST-Sp100, 15 μM SUMO-1, the reactions were initiated by adding 5 mM ATP, or water for the negative control. The reactions were quenched with SDS sample buffer containing 360 mM DTT. Staining was achieved with Simply Blue.

### 1.4 Crystallization, data collection, and structure determination

Search for optimum Ubc9-RWD protein complex growth conditions was conducted using the hanging drop vapor diffusion method at 20 °C using Wizard 3 and 4 crystallization screens (Rigaku Reagents). The complex was prepared by mixing 500 μL of 1 mM RWD and 1 mM Ubc9 solutions (1:1 M ratio). The complex was incubated on ice for 2 h. The solution was spun down at 5000 rpm for 5 min to separate the precipitate. The concentration of the clear protein solution was between 13−18 mg/ml. To make sure we obtained the Ubc9-RWD crystals instead of a single protein of either Ubc9 or RWD, we also screened the single proteins using the same conditions as the complex. Three crystallization conditions (Wizard 3, conditions #12 and #19; and Wizard 4, condition #48) were optimized to produce Ubc9-RWD crystals by varying the buffer pH, protein concentration and PEG concentrations. Crystals of Ubc9-RWD complex displaying a flat plate morphology were obtained after 4−5 days in the optimized condition of 10% (w/v) PEG8000, 100 mM HEPES/NaOH, pH 8.0 and 8% ethylene glycol at a volume ratio of 2:1 Ubc9-RWD complex to crystallization solution. The crystal of the heterotrimeric Ubc9-RWD belonged to space group *P* 1 2_1_ 1 with unit cell dimensions of *a*=63.23 A°;, *b*=34.86 A°, *c*=114.51 A°, *α*=90°, *β*=98.53°, and *γ*=90°. X-ray diffraction data was collected at the X-ray Crystallography Core at City of Hope on Rigaku Micromax−007 HF instrument equipped with R-AXIS IV++ plate reader. The Ubc9-RWD heterotrimer crystals diffracted to 2.7 A°. Diffraction data was reduced and scaled using XDS and XSCALE [15], respectively followed by conversion into mtz format by XDSCONV and F2MTZ [15]. Initial phase information was obtained through molecular replacement using Molrep [16] and the structures of Ubc9 (PDB ID: 1U9B) and human RWD domain (PDB ID: 2EBK). The initial model was improved through iterative refinement using Phenix [17] with model building in Coot [18].

## Figures and Tables

**Fig. 1:**
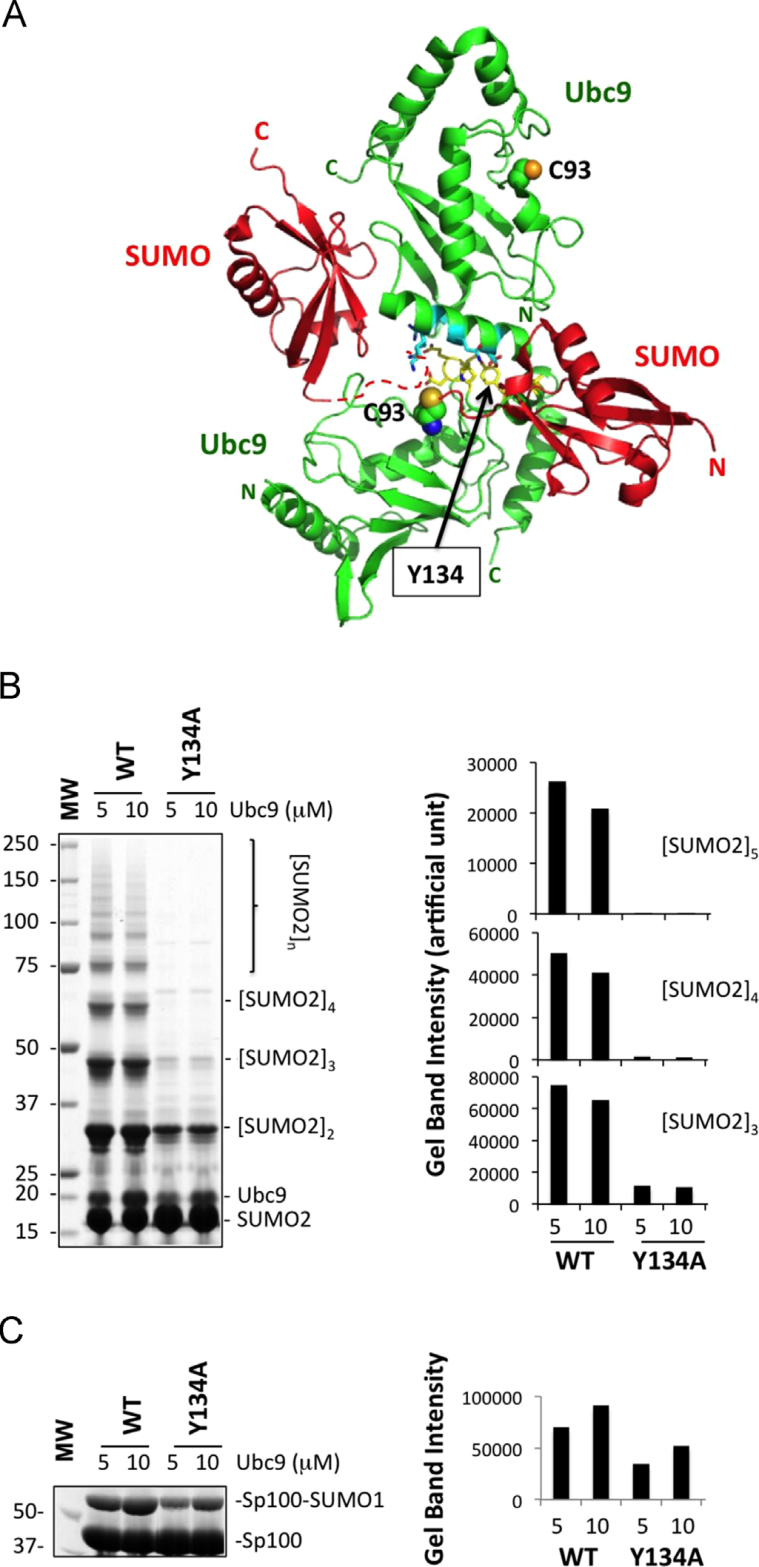
Proposed mechanism of poly-SUMO chain formation. (A) A model of how Ubc9 homodimer could stimulate SUMO-chain formation. The Ubc9 homodimer observed in the crystal structure of the heterotrimer contains two Ubc9 molecules (green) and one RWD (not shown). The residues at the binding interfaces are indicated in cyan (the top Ubc9 molecule) and yellow (the bottom Ubc9 molecule) and with their sidechains shown. The catalytic Cys residue of Ubc9 is shown with spheres. Y134 at the interface is indicated. The structure of non-covalent Ubc9-SUMO complex is superimposed onto the top Ubc9 molecule of the Ubc9 homodimer, resulting in the position of the SUMO molecule, shown in red, on the upper left side. The dashed red line represents the flexible N-terminal segment of SUMO that contains the SUMOylation site but did not have electron density in X-ray diffraction. A hypothetical SUMO molecule that forms a thioester conjugate with the bottom Ubc9 is shown on the lower right side. (B) To test whether the Ubc9 homodimer observed here is important for poly-SUMO chain formation, the Y134A mutant at the Ubc9 homodimer interface was used to test the ability of Ubc9 in stimulating the formation of poly-SUMO chains. Residues at the N-terminal surface of Ubc9 were not mutated, because this surface is directly involved in a higher affinity interaction with E1 for the transfer of SUMO from E1 to E2 than that of surface containing Y134 near the catalytic Cys [7], [12]. SDS-PAGE analysis of poly-SUMO chain formation in the presence of wild-type and Ubc9 Y134A mutant is shown to the left. Quantification of gel band intensity using the ImageJ software is shown to the right. Y134A showed severe defects in catalyzing poly-SUMO-2 chain formation. (C) Because Y134A plays a role both in the transfer of SUMO from E1 to E2, and from E2 to target proteins [13], [14], we examined whether the defects in poly-SUMO-2 chain formation is only due to the effect of the mutation on SUMO transfer from E1-E2 and E2-substrate using mono-SUMO-1 modification of the substrate Sp100. SDS-PAGE analysis of mono-SUMO-1 modification of Sp100 in the presence of wild-type and Ubc9 Y134A mutant is shown to the left. Quantification of gel band intensity using the ImageJ software is shown to the right. The Y134A mutant showed more severe defects in catalyzing poly-SUMO-2 chains than mono-SUMO-1 modification of Sp100, indicating that its defect in catalyzing poly-SUMO-2 chain formation was not only due to its effect on general SUMOylation. These data suggest that the Ubc9 dimer observed in this crystal structure is likely important to the formation of poly-SUMO chains.
